# Adapting online education resources to meet the needs of an emergency medicine training programme in a pilot feasibility study

**DOI:** 10.15694/mep.2021.000080.1

**Published:** 2021-03-26

**Authors:** Sierra Beck, Emma Carlin, Kristen Grabow Moore, Megan Anakin

**Affiliations:** 1 University of Otago; 2 Southern District Health Board; 3 Emory University School of Medicine

**Keywords:** online resources, curriculum, emergency medicine, graduate medical education, feasibility study

## Abstract

This article was migrated. The article was marked as recommended.

**Introduction:** Online resources are available to enhance emergency medicine training programmes. The aim of this study was to evaluate the feasibility of using online case-based resources created in the United States in a New Zealand emergency medicine training programme.

**Methods:** Evaluation data were collected from junior doctors and educators after they participated in the programme. Data sources included research notes and questionnaire responses. The data were analysed qualitatively using a general inductive approach.

**Results:** Evaluation feedback from 19 junior doctors and 14 educators was interpreted to suggest that the online resource, with minor adaptations, was feasible to use in a New Zealand emergency medicine training programme. Findings indicated that educators were able to modify the materials to modify to meet local requirements, however, the opportunity to include a cultural component was missed. Participants appreciated the case-based format and felt that they established a safe and encouraging learning environment with each other. Participants were able to develop a systematic approach to emergency situations and identify red flags related to deteriorating patients.

**Discussion and Conclusion:** Evaluation findings indicate that adapting an online-sourced curriculum is feasible to educators and acceptable to junior doctors and educators participating in a single New Zealand based Emergency Medicine training program. Educators in other international training settings may find the lessons learned helpful when adapting online resources to address the learning needs of their junior doctors. Next steps are to evaluate the impact of this resource on the knowledge and skills learned by junior doctors and any changes in their care for patients in emergency medicine situations.

## Introduction

Emergency medicine training programs are employing a variety of learning strategies beyond traditional lecture-based delivery of education to enhance the engagement of the trainees ( Brazil, 2014; Tan, Brainard and Larkin, 2015). Case-based learning is one learning strategy that requires learners to recall previously covered material and use it to solve cases based on clinical practice ( Williams, 2005). This strategy provides opportunities for teamwork, hypothesis generation, as well as testing and self-evaluation of prior knowledge. Case-based learning is generally acceptable to learners and their instructors, in a variety of settings; however, it can be instructor and resource intensive ( Srinivasan *et al.,* 2007; Garvey, O’Sullivan and Blake, 2000). Instructors may have limited resources for curriculum development, and consequently, they may find it challenging to develop and evaluate resources that meet the needs of a diverse group of postgraduate learners. One way to address this challenge comes from the Free and Open Access Medical Education (FOAM) movement ( Nickson and Cadogan, 2014). The FOAM movement has produced a wealth of online educational resources which instructors can use to augment local training programs. These resources can be freely used with acknowledgement. These resources appear to be acceptable and useful according to junior doctors and their educators, but how to adapt resources to local learning needs is yet to be explored ( Goldman, Barthman and Merritt, 2019). To explore this need, the aim of this study was to evaluate the feasibility of using online case-based resources created in the United States in a New Zealand emergency medicine training programme.

## Methods

An evaluation approach was used in this study ( Creswell and Creswell, 2018). A survey-based study design was approved by departmental review and ethical approval was granted by the University of Otago Human Ethics Committee (D18/280). The implications of this study for Māori were discussed with the Ngāi Tahu Research Consultation Committee. Locality authorization was obtained from the Southern District Health Board to conduct this study from September to December 2018 at the Dunedin Public Hospital, Dunedin, New Zealand.

Participants in this study included junior doctors and their educators. The junior doctors were registrars employed in the Emergency Department at the Dunedin Public Hospital. We use the term junior doctor to refer to someone who has completed their undergraduate medical training such as an MB ChB degree and who has not yet enrolled in a postgraduate training programme. Educators who participated in this study included registrars in the advanced stages of the Fellowship Australasian College of Emergency Medicine (FACEM) training programme and senior emergency medicine doctors who had completed FACEM training and were employed by the Dunedin Public Hospital and/or the University of Otago.

FOAM resource used was the *Foundations of Emergency Medicine* (FoEM) curriculum ( https://foundationsem.com/). FoEM is a free, open-access, case-based, and is used to supplement the American Board of Emergency Medicine curriculum that is used in some emergency medicine training programs in the United States ( Goldman, Barthman and Merritt, 2019). FoEM resources are designed to be used in a flipped classroom manner and targeted to early stage trainees within a postgraduate system. FoEM resources provide a systems‐based review of fundamental knowledge and core diagnoses for emergency medicine. The case-based content was reviewed by instructors prior to delivery to ensure consistency with Dunedin Public Hospital practice such as medication availability, common referral pathways, and laboratory units. During weekly one-hour sessions, junior doctors worked in small groups rotating though three case discussions facilitated by an educator. Notes were kept by the research team about how the educators used and modified the FoEM resources.

Junior doctors were invited to participate when they attended their first structured education session that included the FoEM resources in September 2018. If junior doctors declined to participate in the study, they were informed that there would be no difference in their training. They completed a questionnaire after participating in the programme for three months. Educators were invited to participate before they facilitated a case discussion session during the study period. They completed the educator version of the questionnaire after they facilitated one or more session in the programme. Questionnaire items are shown in Table 1. All participants gave written informed consent prior to participating in the study.

**Table 1.** Questionnaire Instructions and Items Provided to Participants.

**Instructions:** Please write brief responses to the following questions/statements.
**For junior doctors** • What aspects of decision-making do you think you learned during the case discussions? • Please suggest an improvement for your teacher to consider before their next case discussion with you. • Did you prefer these interactive cases or would you prefer to return to lecture based curriculum? • Any other comments?
**For educators** • What aspects of decision-making did you impart to learners during the case discussions? • How did you help learners get the most of out of these case-based discussions? • Do you think trainees learned medical decision making better from lectures or interactive cases? • Please explain why? • Any other comments?

A general inductive approach ( Thomas, 2006) was used to analyse two data sets separately. First, research notes were analysed to identify how the educators used and modified the FoEM resources. Second, questionnaire responses were analysed to identify how all participants described their reactions of the FoEM resources in the training programme. The goal of the coding procedure was to identify a limited number of curriculum features in common in each set of data. Coding was derived from the words or phrases written in the research notes and reflection responses. Only one code was applied to each note or response. Similar codes were grouped until a limited number of themes were identified to represent the curriculum features and reactions in each data set.

## Results/Analysis

Twenty-nine staff members participated in this study. In general, the 15 participating junior doctors were younger, held more junior job types, and had less training than the educators than the 14 educators. More females volunteered to participate (17 female, 12 male) and no participant self-identified their ethnicity as Māori or Pacific Island (15 New Zealand European, 4 Asian, 10 not New Zealand European, Māori, Pacific Island, or Asian).

Findings from the research notes indicated that educators made six modifications to the FoEM curriculum to adapt it to the local context. First, since the FoEM curriculum was created for a postgraduate training programme in the United States, each case required approximately 15 minutes for an educator to review it prior to a session to ensure local relevance and appropriateness. For example, a case on Rocky Mountain Spotted Fever was skipped entirely. Second, laboratory values such as glucose and creatinine required conversion to standard international units. Third, terminology such as ‘primary care provider (PCP)’ required translation to ‘general practitioner’ to be familiar to doctors in New Zealand. Fourth, pharmaceutical recommendations, including antibiotics usage, were reviewed to ensure medications were appropriate to the local context. Fifth, practice differences between our local hospital and the plan of care outlined in the cases were identified and addressed by incorporating discussions of local practice variation. Sixth, there were no Māori or Pacific Island patients in the FoEM cases and this omission was not addressed by the educators.

Findings from the questionnaire responses indicated that educators and junior doctors’ reactions to the modified FoEM curriculum were positive and could be characterised by four themes shown in Table 2. First, participants expressed that the case-based format was acceptable. Second, participants noted the importance of developing a systematic approach to patient care in an emergency medicine context. Third, they noted that learning to identify ‘red flags’ or signs that must not be missed so that they could manage deteriorating patients was also an important feature of the curriculum. Fourth, participants expressed that a safe and challenging learning environment encouraged them to ask and answer questions and be willing to make and accept mistakes.

**Table 2.** Curriculum Features Identified by Participants (n = 29) and Supported with Representative Reflective Responses.

Curriculum Feature	Representative Reflective Response
Case-Based Format	*Prefer interactive* (P11JD) *Interactive cases, they typically fall asleep in lectures* (P26E)
Developing a Systematic Approach	*Systematic approach even when uncertain* (P16JD) *Prioritisation, systematic approach, when to seek help* (P17E)
Identifying Red Flags and Managing Deteriorating Patients	*Interventions - timing, acute life threatening care* (P13JD) *Prioritisation and recognition of red flags, and how this can influence decisions* (P29E)
Encourage a Safe and Challenging Learning Environment	*All the registrars and house officers to extract the history rather than [educators] telling us* (P10JD) *Encourage interaction in nonthreatening small groups, encourage all to take part* (P28E)

Note. Abbreviations used: P = participant, JD = junior doctor, E = educators.

## Discussion

Adapting internationally sourced FOAM resources for use in a New Zealand emergency medicine training programme appears to be feasible. Adapting the FoEM resources required several modifications that should be considered by others interested in using internationally sourced FOAM resources. The modifications included skipping cases that are not relevant to the local health care context, converting laboratory values to standard international units, translating terminology, review pharmaceutical recommendations, researching and discussing patient management to align with local hospital practices. While these differences may be viewed as a drawback, educators used them as opportunities to further explore factors that may impact a doctor’s ability to prioritise and to make decisions effectively, efficiently, and safely. One element noticed but not addressed by educators in this study was the omission of cultural competency and cultural representation because there were no Māori or Pacific Island patients in the FoEM cases. The case-based learning format may be well suited to educational goals set out in Manaaki Mana ( Australasian Association for Emergency Medicine, 2019) and to address dimensions health valued by Pacific Island peoples ( Pulotu-Endermann, 2001). Incorporating a cultural perspective should be part of the modifications of the FoEM resources for future sessions to ensure the health equity needs of Māori and Pacific Island patients are met by doctors in the FACEM training program.

A curriculum feature identified by all participants involved developing a systematic approach that includes attending to red flags when managing a deteriorating patient. This finding underscores the importance of learning prioritisation and decision-making skills in local training programmes to standards established by the Australasian Association for Emergency Medicine (2015; 2020). Another curriculum feature appreciated by all participants was the need to establish a safe and challenging learning environment. This finding is well-supported by evidence and pedagogy developed to maintain psychological safety while encouraging learners to set challenging goals for themselves ( Dumont, Istance and Benavides, 2010).

Since a focus of this study was to identify challenges faced by educators when implementing an external training curriculum, the design of this study focused the reactions of the participants. This focus on participant reactions resulted in limiting the data collection and evidence interpretion to the lowest level of Kirkpatrick’s framework for programme evaluation ( Praslova, 2010). Next steps will be to design a study that gathers higher quality data to evaluate program outcomes such as a change in knowledge, skills, behavior, and impact on clinical outcomes.

## Conclusion

This study’s findings were interpreted to suggest that adapting an online sourced curriculum to an international training environment was feasible and acceptable to participating junior doctors and educators in a single New Zealand based emergency medicine training program. Educators were able to modify the online resources to meet local requirements, however, the opportunity to include a cultural component was missed. Features of the curriculum appreciated by junior doctors and educators in this training programme included the case-based format, the opportunity to develop a systematic approach that includes attending to red flags when managing a deteriorating patient, and the need to establish a safe and challenging learning environment.

## Take Home Messages

• Online resources are available to enhance emergency medicine training programmes but they may need to be adapted to meet local needs.
• Modifications to online resources were feasible and acceptable to junior doctors and educators in a New Zealand emergency medicine training context.
• When adapting online resources to meet local expectations, educators in other contexts may need to skip cases that are not relevant to the local health care context, convert laboratory values to standard international units, translate terminology, review pharmaceutical recommendations, and research patient management so it aligns with local hospital practices.

## Notes On Contributors

Sierra Beck, FACEM, Senior Lecturer, Department of Medicine, Otago Medical School - Dunedin Campus, University of Otago, Dunedin, New Zealand.

Emma Carlin, Registrar, Dunedin Hospital, Southern District Health Board, Dunedin, New Zealand.

Kristen Grabow Moore, MD, MEd, Assistant Professor, Emory University School of Medicine, Atlanta, GA, USA.

Dr Megan Anakin, BSc (McGill), BFA (NSCAD), BEd (Dalhousie), MEd (Simon Fraser), PhD (Otago), AFANZAHPE, Lecturer and Education Adviser, Education Unit, Otago Medical School - Dunedin Campus, University of Otago, Dunedin, New Zealand. ORCID ID: https://orcid.org/0000-0002-6499-7802

## Declarations

For transparency, Megan Anakin is a member of the MedEdPublish Editorial Board.
