# Supplementary material for: Adapting online education resources to meet the needs of an emergency medicine training programme in a pilot feasibility study
Source: MedEdPublish (2016). 2021 Mar 26;10:80. [Version 1] doi: 10.15694/mep.2021.000080.1 (PMC10939642; doi:10.15694/mep.2021.000080.1)
Supplement: Peer review history

# Reviewer report: Reviewer response for version 1

**DOI:** 10.21956/mep.18959.r27139

**Published:** 2021-05-27

**Authors:** Trevor Gibbs

**Affiliations:** 1 AMEE

This review has been migrated. The reviewer awarded 5 stars out of 5 I enjoyed reading and reviewing this paper and particularly appreciated the final take home messages which are wholly applicable to the present day teaching and learning situation. I found the paper to be well-written, very clear in its purpose and extremely clear in its conclusions and take home messages.One sees many situations where teaching resources are transported and implemented from one country to another without evaluation. I must admit my thoughts are that like a transplant, many of the transplantations are eventually rejected. This paper showed that with contextual modifications, then moving methodologies from one country to another is possible and can produce a positive effect.The paper is very specific to one particular organisation and I , like my co-reviewer, would have valued a comparative experience from another organisation to see which features / cases they would have differed on. However this is a little bit of "icing on the cake" and do not think it detracts too much from a very informative paper.I fully recommend this paper to all involved in curriculum planning and I look forward to the follow-up paper describing the longer term evaluation of the activity.

Reviewer Expertise:

NA

No decision status is available

# Reviewer report: Reviewer response for version 1

**DOI:** 10.21956/mep.18959.r27138

**Published:** 2021-03-26

**Authors:** Barbara Jennings

**Affiliations:** 1 Norwich Medical School

This review has been migrated. The reviewer awarded 4 stars out of 5 Thank you to the authors for this well written paper about participant (educator and trainee) reaction to an adapted online curriculum for emergency medicine training. It raises the important issue of free & open access medical education (FOAM) and is a timely reminder that not all teaching materials have to be created from scratch, there is a wealth of excellent resources that can be shared and reused. Although I am in a different field, it strikes me from #mededtwitter that emergency medicine is a leader in FOAM.There are many important points to recommend the structure and content of the paper: a title and abstract that succinctly and accurately describe the study and its findings; the research governance including the REC approval details and the explanation of consenting in the methods section; the explicit description of the research method used. In the results section, curriculum modifications were noted and challenges e.g. full consideration of cultural competence as well as the benefits of a case based and clinically systematic curriculum.The article is very clearly written with a logical flow of information between the methods-results- discussion and conclusion, so I only had one unanswered question by the time I had finished reading it. This was about the items selected from the Foundations of Emergency Medicine (FoEM) curriculum (https://foundationsem.com/) – do the authors think it could be helpful to readers if they itemise the particular resources selected? Or perhaps the resources are best matched to the host needs (e.g. ensuring consistency with Dunedin Public Hospital practice in this instance) and so a precise list may be unlikely to be generalisable to other centres?

Reviewer Expertise:

NA

No decision status is available
